# Mapping the Dynamic Functions and Structural Features of AcrB Efflux Pump Transporter Using Accelerated Molecular Dynamics Simulations

**DOI:** 10.1038/s41598-018-28531-6

**Published:** 2018-07-11

**Authors:** Shirin Jamshidi, J. Mark Sutton, Khondaker Miraz Rahman

**Affiliations:** 1 School of Cancer and Pharmaceutical Science, King’s College London, London, SE1 9NH UK; 2 Public Health England, National Infection Service, Porton Down, Salisbury, Wiltshire SP4 0JG UK

**Keywords:** Computational models, Antimicrobial resistance

## Abstract

Multidrug efflux pumps confer resistance to their bacterial hosts by pumping out a diverse range of compounds, including most antibiotics. Being more familiar with the details of functional dynamics and conformations of these types of pumps could help in discovering approaches to stop them functioning properly. Computational approaches, particularly conventional molecular dynamics simulations followed by diverse post simulation analysis, are powerful methods that help researchers by opening a new window to study phenomena that are not detectable in as much detail *in vitro* or *in vivo* as they are *in silico*. In this study, accelerated molecular dynamics simulations were applied to study the dynamics of AcrB efflux pump transporters in interaction with PAβN and tetracycline as an inhibitor and a substrate, respectively, to compare the differences in the dynamics and consequently the mechanism of action of the pump. The different dynamics for PAβN -bound form of AcrB compared to the TET-bound form is likely to affect the rotating mechanism typically observed for AcrB transporter. This shows the dynamics of the active AcrB transporter is different in a substrate-bound state compared to an inhibitor-bound state. This advances our knowledge and helps to unravel the mechanism of tripartite efflux pumps.

## Introduction

Overexpression of the resistance nodulation cell division (RND) type of tripartite efflux pumps in the Gram-negative pathogens is a major component of multidrug resistance (MDR)^1^. These types of efflux pumps recognize a diverse range of compounds and pump them out from the bacteria cell via proton-motive force (PMF) secondary transporters like AcrB, part of AcrAB-TolC efflux pump, present in *Klebsiella pneumoniae and Escherichia coli*^2–8^.

**Figure 1:**
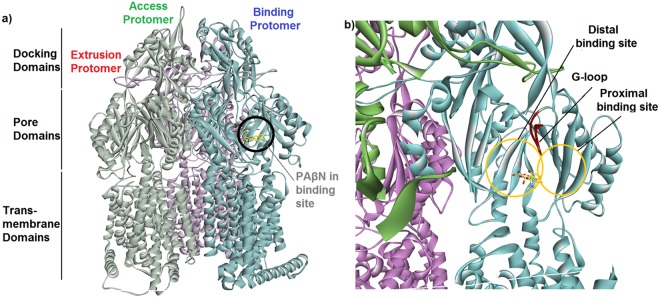
(**a**) The proposed homotrimer model structure of the AcrB efflux pump transporter in complex with PAβN. It represents the structure that was obtained after carrying out the homology modelling, minimization and equilibration. The full-space complete homotrimer structure of AcrB has been shown on the right side of the picture, and each subunit has been represented by different colours; The binding site in binding monomers determined by SMINA molecular docking somewhere close to the distal pocket has been represented by a black circle (**b**) Tetracycline in the multi-binding sites within the binding protomer of the AcrB transporter.

The substrate specificity and selectivity of AcrAB-TolC efflux pump depends on the homotrimer structure of AcrB, with threefold asymmetric conformation, in which each monomer adopts a different conformation, access, binding and extrusion^5,9–14^. AcrB of *K*. *pneumoniae*, that has similar sequence to the solved crystal structure of AcrB of *E*. *coli*, pumps out the compounds employing a rotating mechanism that is allosterically coupled, in which each monomer successively adopts one of the three conformations^10,11,14–20^. Recent computational works have further shed lights on the relationship between functional rotation and substrate transport^21–23^. Structures with bound drugs revealed two discrete multisite binding pockets separated by a switch loop, with the distal pocket in the binding (tight) state and a proximal pocket in the structure^24,25^ (Fig. 1). The proximal and distal binding pockets that are involved in forming the multibinding site of AcrB, as the substrate-selective part of the pump, play key roles in its specificity and binding to substrates^2,24–32^. The G-loop controls the access of substrates to the distal pocket by forming a boundary between the proximal and distal binding pockets^2^. Under the G-loop, there is a narrow channel that connects proximal and distal pockets to each other (Fig. 1). The pockets are enriched in aromatic, polar and charged amino acid residues that form favourable interactions with the transported substrates. The microenvironment of the distal binding pocket within AcrB has been studied in detail^33,34^. Since the distal binding pocket includes many hydrophobic, polar and charged residues, this microenvironment mediates extrusion of a wide range of compounds by AcrB^4,35,36^. Based on the multisite-drug-oscillation hypothesis by Yamaguchi^8^, the spacious multidrug-binding pocket may have numerous binding sites even for a single substrate, suggesting that substrates may move between binding sites during transport. This hypothesis could explain the apparently broad substrate specificity of cell membrane exporters and their highly efficient ejection of drugs from the bacteria cells.

Substrates that are taken up from the entrance ports, in particular the cleft, of an efflux pump could be transported through dual multidrug-binding pockets by a peristaltic mechanism at the substrate translocation channel^37^ through both proximal and distal pockets, and could be potentially extruded from the top exit port. Actually, the asymmetric trimer is supportive of a rotating access mechanism for drug binding and release (Fig. S1).

Designing effective inhibitors to stop these types of pumps from effluxing out antibiotics requires an understanding of the functional dynamics and molecular mechanisms of conformational changes of this type of protein. There are only a few solved structures for AcrB of *E*. *coli* in complex with different compounds^10,20,25,38^ and no structure for AcrB of *K*. *pneumoniae*.

Conventional molecular dynamics (cMD) simulations enable simulations on the order of tens to hundreds of nanoseconds; however, longer simulations are required to monitor biological processes that typically occur on longer time scales of up to milliseconds or more^39^. Recently developed advanced sampling techniques such as accelerated molecular dynamics (aMD) simulations allows access to these longer time scale molecular events beyond those obtainable with cMD^40,41^. aMD reduces the height of local barriers which allows faster calculations and time efficient simulation of biomacromolecules^42^. It simplifies the sampling by requiring only the evolution of a single copy of the system and does not require any previous knowledge of the shape of the potential energy profile^39^.

aMD simulations allow determination of time-dependent protein conformational changes which enables sampling the conformational space more efficiently than conventional molecular dynamics simulations^43–48^ and has been fully integrated into commonly used software such as Amber^39,49^. Advanced sampling techniques employed by aMD extend the time scale of MD simulations that converges the correct canonical probability distribution and enables rapid sampling of the conformational space^42^.

**Figure 2:**
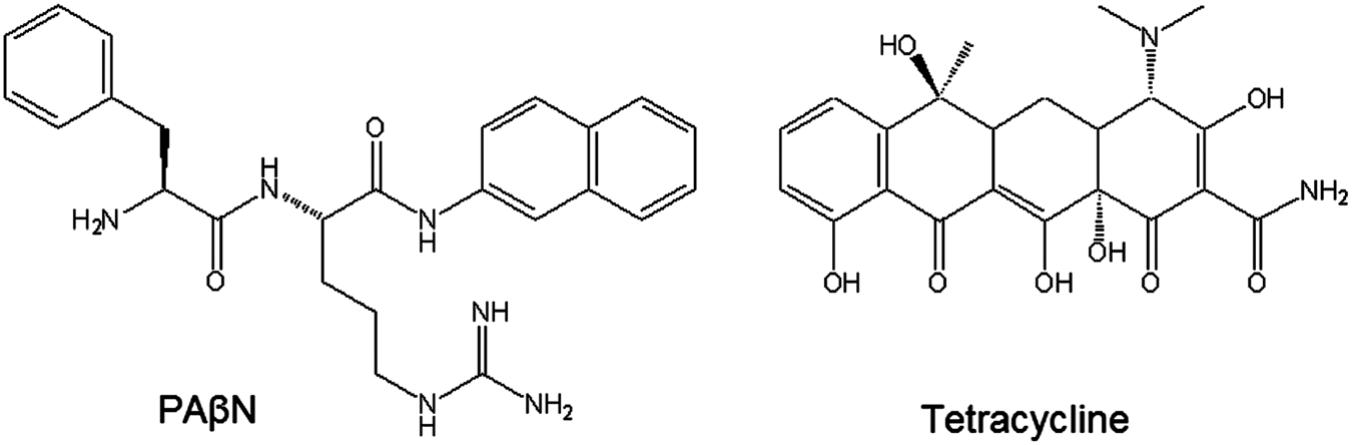
Chemical structures of the compounds, PAβN and Tetracycline, used in this study.

Nikaido *et al*. in their recent works^2,50,51^ studied a truncated model of AcrB transporter from *E*. *coli* in interaction with different ligands by cMD simulations. This showed the important role of the G- loop and the deep hydrophobic groove of the distal site, indicating that the binding mode of compounds to the transporter alters the efflux of other substrates. Similarly, Fischer and Kandt studied the conformational changes in the porter domain of AcrB from *E*. *Coli* conventional molecular dynamics^52^. In this study, we use the sampling power of aMD simulations to reveal the time-dependent conformational changes in the homology-modelled multidrug efflux pump AcrB from *K*. *pneumoniae* (SI), in interaction with Phenylalanyl-arginine-β-naphthylamide (PAβN), an inhibitor and modulator of AcrB^7,53^, and tetracycline (TET), a substrate for the transporter (Fig. 2) after generating the starting complexes by molecular docking. Principal component analysis (PCA) has been used to investigate the correlations among various important regions of the complexes in the course of aMD simulations and to distinguish between different conformational states.

## Results and Discussion

### Structures of the complexes

**Figure 3:**
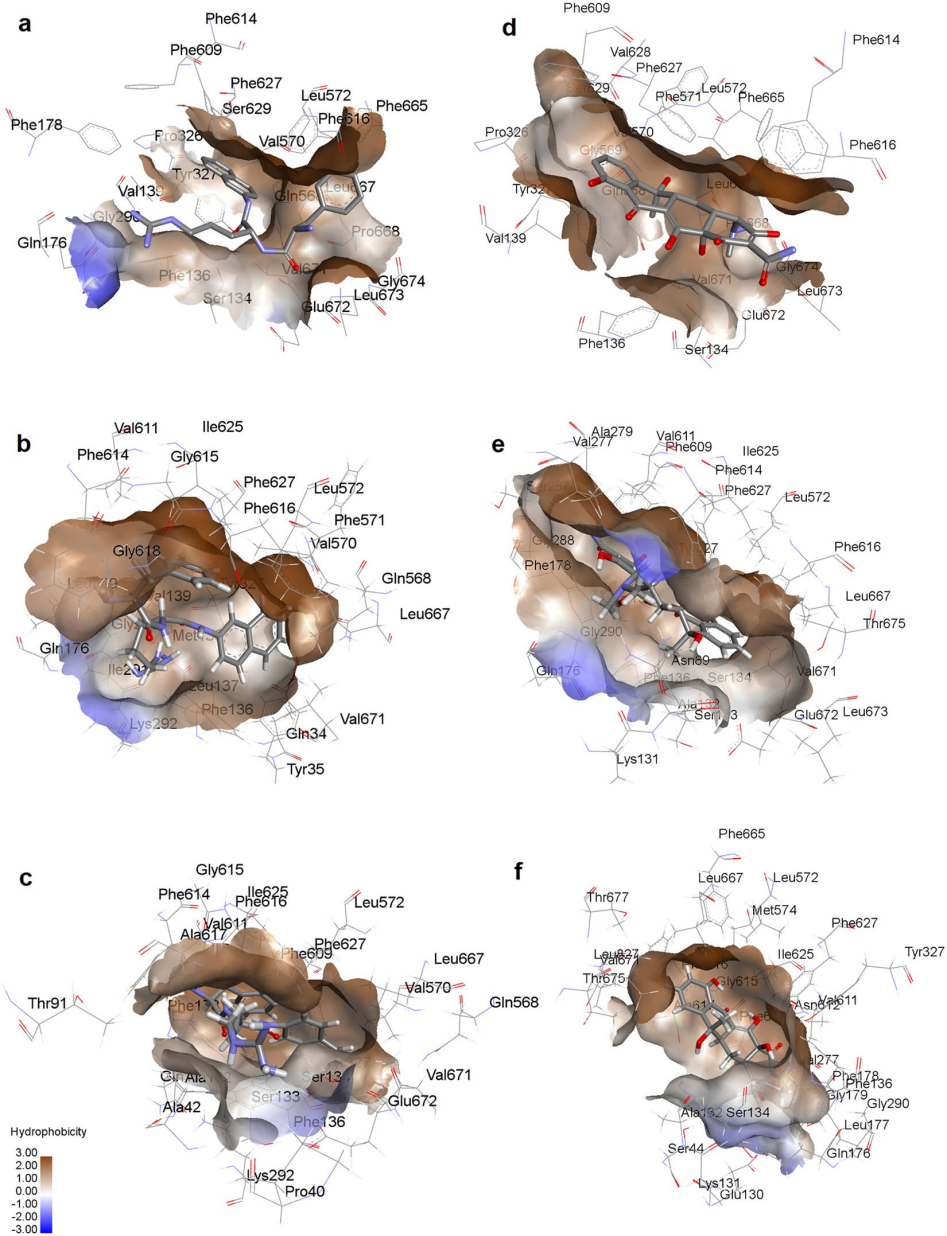
3D structures of PAβN (right column) and TET (left column) in the multi binding site of AcrB; (**a** & **d**) after GOLD molecular docking, (**b** & **e**) average structure after 100 ns cMD and (**c** & **f**) average structure after 200 ns aMD.

Figure 1 shows the generated structure and important parts of AcrB transporter for *Klebsiella pneumoniae* by homology modelling using Swiss Model webserver with sequence identity 91.6% against the template, crystal structure of AcrB of Escherichia coli (PDB id code 4DX5). The trimer of the AcrB protein was obtained as the final model from homology modelling in a 3D PDB structure format. The template that was used for the homology modelling was a monomer structure and, therefore, the generated model was also a monomer structure. The assembly procedure was performed using the Accelrys discovery studio. The accuracy and validity of the generated AcrB model was examined in detail, and was shown in the different panels of Fig. S2. According to the SMINA molecular docking results, the location for the binding of PAβN to the protein structure was identified in the multisite binding pocket within the binding protomer of the transporter (Fig. 3). The docked complex of PAβN-AcrB was comparable to the previous structures determined for ligand-bound AcrB by Nakashima *et al*.^24^. Only one PAβN was bound to the binding monomer of homotrimer, and it was bound only to the multi-binding site. Also, other favorable docked poses showed that PAβN could bind to the access monomer of the AcrB homotrimer, which suggests that PAβN could be forced to pass through the path, during the dynamic of the efflux process, by a transient conformational change from the access form to the binding form, and PAβN would move to the gate of the distal pocket in the binding state. There were strong hydrophobic interactions between PAβN and Phe616 of AcrB which is located at the tip of the hairpin-like G-loop and forms a partition between the proximal binding pocket and distal binding pocket at the top of the channel between the two pockets. GOLD molecular docking of PAβN and TET to the located binding site by SMINA also showed that the affinity of PAβN to the AcrB transporter (binding energy -39.9 kcal/mol and score 37.3) is much more favourable over TET (binding energy -25.6 kcal/mol and score 22.2). Phe-cluster residues, including Phe136, Phe178, Phe616, Phe627 and Phe665, provided effective Pi interactions between the ligand and the transporter. These strong interactions resulted in higher score and favourable docking energy.

### Important regions of AcrB involved in interaction with ligands

Table 1 shows the residues of AcrB that interact with the ligand during the course of the simulation. They have been marked by sequence alignment of AcrB *K*. *pneumoniae* to the solved-structure of AcrB from *E*. *coli*^9,51^. The matched amino acids in the alignment of AcrB sequences from *E*.*coli* and *K*. *pneumoniae* occupy the same locations in their corresponding PDB structures.

**Table 1:** The key residues, forming different important regions of AcrB involved in interaction with ligands or functional dynamics of the pump.

Region	*K*. *pneumoniae* AcrB Lining residues
Proximal binding site	S79, T91, S134, S135, K292, L572, M574, Q576, F616, T623, M661, F663, F665, N666, L667, L673, T675, D680, R716, N718, E825
Distal binding site	S46, Q89, S128, E130, S134, F136, Q176, L177, F178, S180, E273, N274, D276, I277, G290, Y327, L572, F609, V611, F614, F616, R619, F627
Cleft	F663, F665, L667, R716, L827
G-loop/Phe-loop	G615, F616, A617, G618
G-loop tip	Phe616
Postulate gate	Q124, Y757

Numbering is according to the amino acids’ positions in the *K*. *pneumonia*.

### Comparative binding of inhibitor and substrate

Molecular docking showed that PAβN was bound to the narrow channel under the G-loop. The naphthylamine moiety bonded to the bottom area of the distal binding site in interaction with Phe627. On the other side of the molecule, the arginine group was in the distal binding site in interaction with Gln176, with the phenylalanine group in interaction with Leu667 of proximal binding site (Figs 3a and S3a). Tetracycline was in interaction with Ser134, a shared residue between the proximal and distal site, at the bottom of the narrow channel of the multi-binding site and mostly toward the distal pocket (Figs 3d and S3d).

The average structure extracted from cMD simulations showed that the naphthylamine moiety, phenylalanine and arginine groups of PAβN were bound to Phe136, Ph614 and Lys292 respectively, all in the distal binding site (Figs 3b and S3b). It appeared that PAβN stayed in the hydrophobic site of the distal pocket. In contrast, tetracycline was found in interaction with Phe614, Tyr327 of the distal binding site and Phe616 of G-loop between the distal and proximal sites (Figs 3e and S3e), which means the antibiotic failed to stay in the hydrophobic pocket.

The monitoring of the interaction energy between the ligands and the amino acid residues of multi-binding site in AcrB, during the course of cMD by extracting the structures every 10 ns, showed that the stability of PAβN-AcrB complex is higher compared to tetracycline-AcrB complex., There are at least three responsive amino acid Phe136, Phe616 and Phe627 with favorable interaction energy that could be observed in all 10 ns panels, whereas there is just one common responsive residue Phe614 during the cMD in the case of tetracycline (Table S1).

At the end of the simulation, the results in the average structure extracted from aMD simulations indicated that the naphthylamine moiety of PAβN was still in interaction with Tyr327 and the phenylalanine moiety was in contact with Phe616 and Phe178 of the hydrophobic groove, in the distal binding site (Figs 3c and S3c). Tetracycline, which was bound to a residue of the distal pocket, Phe614, was mostly in interaction with the residues of G-loop, Gly615, Phe616 and Ala617, as well as a residue from the proximal site, Leu667 (Fig. 3f and S3f). This data suggests that PAβN could effectively interact with the hydrophobic groove of the distal pocket, as expected of a potential efflux pump inhibitor, which, by peripherally binding to the hydrophobic trap, could occlude the passageway and consequently interfere with the binding of the other compounds^2,50,51^. On the other hand, tetracycline formed weaker interactions with the hydrophobic pocket during the course of the simulation, which would eventually result in efflux through the transporter. This results in a good agreement with what Kinana and coworkers showed regarding the effects of various amino acids of binding pocket in substrate binding^54^. The monitoring of the interaction energies between the ligands and the key amino acid residues of multi-binding site in AcrB, during the course of aMD by extracting the average structures (Table S2), showed that the stability of PAβN-AcrB complex is higher compared to TET-AcrB complex. There were three amino acids that formed favourable interactions with PAβN compared to a single amino acid that interacted with Tetracycline during the course of the simulation (Table S3 & S4).

Monitoring the distances between the ligands and some of the key residues of the multi-binding site of AcrB (Fig. S4) showed that, in the case of PAβN, the distances reach a steady state very soon via tight binding of the ligand to the deep hydrophobic groove, but for tetracycline they are fluctuating and variable, which means the ligand formed transient interactions moving around the multi-binding site. These results are in a good agreement with the multisite-drug-oscillation hypothesis Yamaguchi and co-workers for AcrB^8^. According to this hypothesis, the substrate is oscillating and may be just occluded in the distal pocket without a specific binding site, as we observed that tetracycline oscillates in the AcrB multibinding site in the spacious drug binding pocket. Also, it can explain the difference in the drug specificity in a certain exporter like AcrB, where tetracycline is exported and PAβN is not exported.

### Binding free energy

cMD simulations followed by calculation of the relative binding free energy of the inhibitor and substrate. Table 2 summarizes the calculated values of the different energy contributions to the relative binding free energy. The calculated binding free energy of tetracycline −11 kcal/mol is notably weaker than PAβN with −28 kcal/mol. The GB approach that resulted in a slightly lower binding energy for both substrate and inhibitor still suggests a more favorable complex in PAβN-bound state. It implies PAβN formed a stable complex that could inhibit the transporter, and that tetracycline is highly likely to end up being pumped out due to the weaker interaction.

**Table 2:** Calculated energy contributions to form the AcrB–PAβN and AcrB–TET complexes (kcal/mol) and inhibition constants (K_d_ in Molar) with standard errors of the mean (in parentheses) after cMD.

Energy distributions	AcrB-PAβN	AcrB-TET
ΔE_ele_	−16.7 (2.1)	−20.1 (2.2)
ΔE_vdw_	−49.2 (2.0)	−41.6 (1.1)
ΔE_sol_	37.8 (3.3)	49.7 (3.5)
ΔG_PB_	−28.1 (2.9)	−11.9 (1.9)
ΔG_GB_	−35.9 (2.3)	−16.3 (2.3)
−TΔS	18.7	18.9
ΔG_bind_	−9.4 (0.7)	6.9 (0.5)
K_d_*	1.4 × 10^−7^	1.2 × 10^5^
K_d_ (Bulk)^**^	8.4 × 10^17^	7.3 × 10^29^

*K_d_ obtained by using ΔG = RT ln K_d_ formula.

**Calculated by considering Avogadro’s number.

The entropy contribution in ligand binding was calculated to determine the absolute binding free energy and dissociation constant for the systems. The calculated values clearly show that the tendency of tetracycline is to be dissociated from the complex, but that PAβN is likely to remain as a complex with AcrB owing to its favourable free energy of binding. Also, comparison of the value of K_d_ in interaction of PAβN with AcrB (1.4 × 10^−7^) and AdeB (4.9 × 10^−6^) in our recent study^55^ showed more binding strength and affinity of the inhibitor to the AcrB transporter over AdeB. This suggests that PAβN is an effective inhibitor of AcrB and explains why it does not inhibit the AdeB efflux pump.

### Principal component analysis

After running aMD simulations, PCA was performed to characterize the conformational transitions in the AcrB transporter, modulated by different ligands, with different roles as the potent inhibitor and substrate. The dendrogram data (Fig. S5) appeared to fall into six and seven clusters for PAβN- and TET- complexes, respectively, and the PCA plots are coloured to show this. By considering six and seven clusters in PAβN- and TET- complexes respectively, it can be seen that AcrB in complex with inhibitor has different dynamics and functions compared to the AcrB in complex with the substrate.

**Figure 4:**
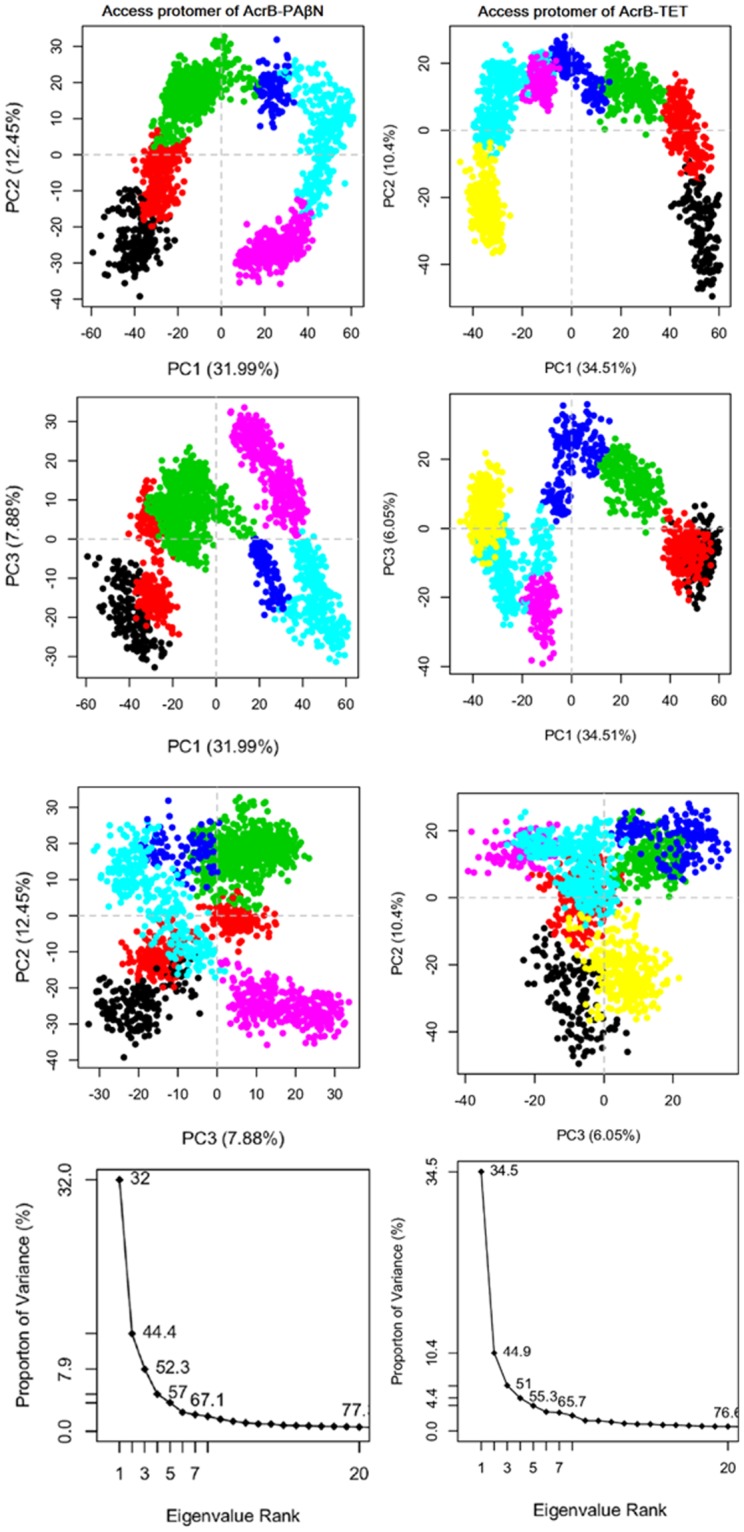
Row (1–3) Conformer plot of PCA data colored by cluster after calculation of cluster groups in the access protomer of AcrB-PAβN (left panels) and AcrB-TET (right panels); Row (4). The rank ordering of the eigenvalues of the covariance matrix. Eigenvalue spectrum: Results obtained from diagonalization of the atomic displacement correlation matrix of Cα atom coordinates from the first snapshot structures. Inset shows histograms for the projection of the distribution of structures onto the first six principal components.

**Figure 5:**
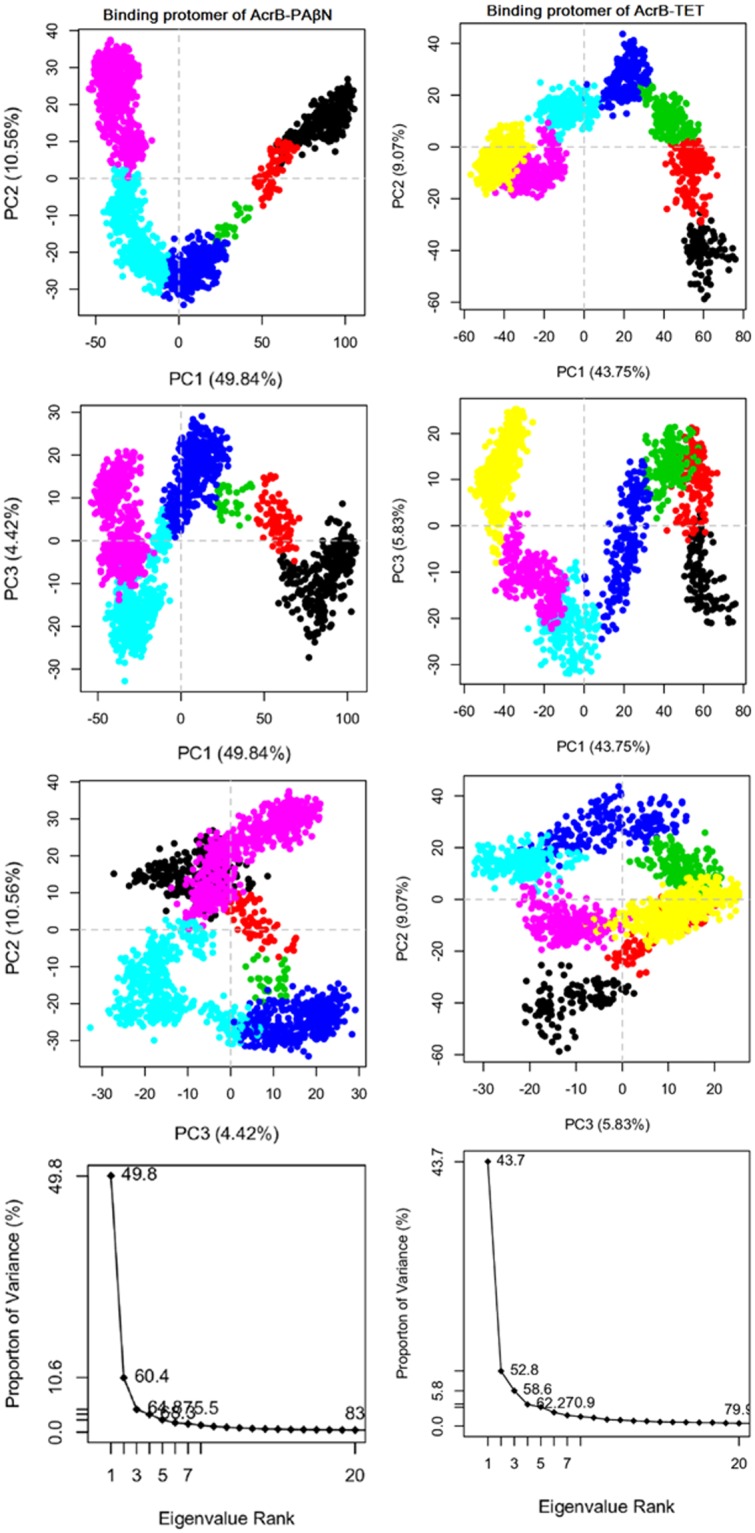
Row (1–3) Conformer plot of PCA data colored by cluster after calculation of cluster groups in binding protomer of AcrB-PAβN (left panels) and AcrB-TET (right panels); Row (4) The rank ordering of the eigenvalues of the covariance matrix. Eigenvalue spectrum: Results obtained from diagonalization of the atomic displacement correlation matrix of Cα atom coordinates from the first snapshot structures. Inset shows histograms for the projection of the distribution of structures onto the first six principal components.

**Figure 6:**
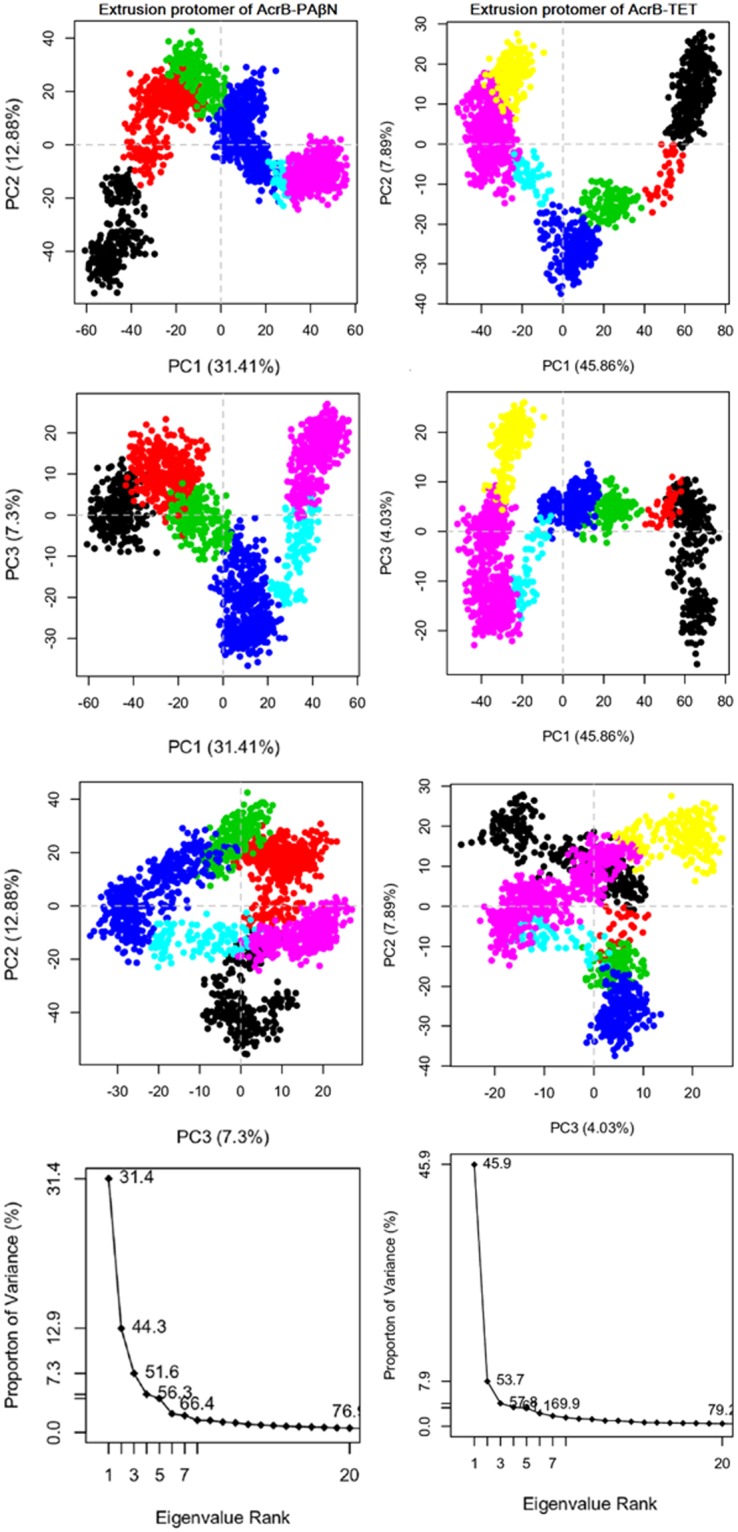
Row (1–3) Conformer plot of PCA data colored by cluster after calculation of cluster groups in extrusion protomer of AcrB-PAβN (left panels) and AcrB-TET (right panels); Row (4) The rank ordering of the eigenvalues of the covariance matrix. Eigenvalue spectrum: Results obtained from diagonalization of the atomic displacement correlation matrix of Cα atom coordinates from the first snapshot structures. Inset shows histograms for the projection of the distribution of structures onto the first six principal components.

Figures 4–6 show conformer plots that display the relationships between different conformers, highlighting the major differences between structures and enabling the interpretation and characterization of multiple inter-conformer relationships. They have been generated in three different panels for diverse protomers in AcrB; Access (loose)/Binding (tight)/Extrusion (open) subunits. PCA highlights that PAβN- and TET-bound conformations are not only different in the binding protomer (Fig. 5) but also that they are completely diverse in their access and extrusion conformations (Figs 4 and 6). In addition, substrate-bound structures are different from inhibitor-bound ones, in general, as they possess diverse conformer graphs with diverse conformational clustering.

The last row in the conformer graphs of Figs 4–6 is the eigenvalue spectrum obtained from the principal component analysis of the computationally determined conformers. The magnitude of each eigenvalue is expressed as the proportion of the total variance (mean-square fluctuation) captured by the corresponding eigenvector. Labels on each point indicate the cumulative sum of variance accounted for by a particular eigenvector and its preceding eigenvectors.

The fluctuations captured by the first principal component were visualized as a trajectory for each complex. They were generated as movies in PyMOL visualizer software for PAβN-bound (Movie S1) and TET-bound (Movie S2) transporters. The movies show a different motion in AcrB transporter in interaction with different compounds applied in this study. There is another twist in the transmembrane domain of PAβN-bound transporter compared to TET-bound transporter, so the observed motion in AcrB-TET complex is not present in AcrB- PAβN complex. Also, movies S3 and S4 were generated to show the dynamics of the AcrB pump in interaction with PAβN and TET respectively using extracted snapshots during the aMD simulations by VMD and movies were created by VideoMach software.

## Discussion

We examined the binding of PAβN and tetracycline to the multi binding pocket of the binding protomer of AcrB, at first by blind docking with the program Autodock SMINA and then using GOLD^56^ flexible molecular docking in the SMINA-located site. The study was followed by conventional molecular dynamics simulations combined by accelerated molecular dynamics, for a whole model of AcrB transporter used to study the dynamics of all the three protomers of the pump.

The accurate computational observation and direct assessment of functionally important protein dynamics and conformations, without any timescale limitations, were performed using an accelerated molecular dynamics simulation approach, by simulating the transition between minima of different potential energy. By this approach, we have gained insight to the comparative function of AcrB in interaction with its substrate or inhibitor. The results show that the motion of the transporter was induced by the ligand in the interaction with the multi-binding site. The induced motion is ligand-dependent, which means it is different for the two bound compounds. The different dynamics for PAβN-bound form of AcrB compared to the Tet-bound form is likely to affect the rotating mechanism typically observed for AcrB transporter. This type of motion dependency has implications for understanding the ligand binding mechanisms in the AcrB tripartite efflux pump.

In agreement with Nikaido *et al*., whose work showed that the binding position of the naphthylamine moiety of PAβN is the reason why it produces efflux inhibition^50,51^, the results of this study showed a strong interaction between naphthylamine moiety of PAβN and the hydrophobic trap of distal binding site. Nikaido *et al*. also suggested that the largest fraction of the binding energy comes from the trap and PAβN interacts strongly with the trap^51^. Similarly, binding free energy calculations showed a very favorable free energy of binding for PAβN in interaction with AcrB. In addition, monitoring of the interaction energy between this inhibitor and key residues of binding site indicate that Ala132, Ala617, Ser134, Phe627, Phe136 and Phe616 in AcrB-PAβN complex with the most favorable energy of interaction mostly belong to the hydrophobic trap.

Nikaido *et al*. computationally showed that PAβN as an inhibitor of AcrB from *E*. *coli* tends to move out the distal pocket at least partially, getting into contact with G-loop, and is thought to control the access of substrates to distal pocket^2^. In another study they showed that the mode of binding of PAβN to AcrB affects the efflux of other compounds^50^. In addition, they experimentally and computationally showed that PAβN inhibited the efflux of other drugs by binding to the hydrophobic trap of the distal binding pocket, and also by interfering with the binding of other drug substrates to the upper part of the binding pocket. Our results also showed that PAβN occludes the channel by sticking to the hydrophobic binding pocket and interaction with other residues within the multi-binding site. Occlusion of the binding protomer affects the regular dynamics and allosteric switching motions of three protomer of the transporter. Consequently, normal rotatingmotion which leads to the extrusion of the compound could be corrupted. Abnormality in the motion of the transporter causes dramatic changes to the pump’s compound extrusion ability. The new dominant dynamics induced by the interaction of the inhibitor with the transporter, by blocking the binding monomer, corrupt the switching of the functional dynamics and prevent the transporter from extruding the compounds. It seems that the efflux pump works by a ligand-dependent dynamics approach that fully affects the functional features of the transporter. Another study on AcrB of *E*. *coli* showed that the assembly of the RND-type efflux systems is dynamically regulated in response to external stimuli^57^.

This study showed AcrB could adopt different dynamics when it interacts with its substrate Tetracycline compared to the inhibitor PAβN-bound form. The simulation data suggests the correlations among the distal and proximal pockets of the multi-binding site, Phe cluster and cleft during the inhibitor binding event could result in the inhibition of the rotating mechanism and prevent the exporter from extruding any other substrates, leading to the inhibition of the tripartite pump. The information obtained from the study will contribute to the design of new, effective and selective efflux pump inhibitors that could play key roles in interrupting the rotating mechanism and allosteric conformational changes that could reverse antimicrobial resistance^2^.

## Methods

cMD and aMD simulations were performed using the program AMBER 12^58^. MD simulations started from the docked structures, obtained from blind docking by AutoDock SMINA^59^, which followed by flexible docking using the GOLD^56^ program. MD simulations of efflux pump complexes have been carried out without the lipid bilayer due to the size of the efflux pump systems. Vargiu^2^ and Kinana^50^ showed that the results for systems with and without the lipid bilayer are essentially the same and equally valid. However, the transporter was not truncated and the whole trimeric protein structure was used in this study. In this study, the power of GPU acceleration combined with the sampling power of aMD raising minima, were used to study conformational transitions that occur in AcrB in interaction with substrate and inhibitor, separately. Also, the CUDA implementation of PMEMD was used to carry out the simulations on GPUs.

### Generating the structures

The structure of the *Klebsiella pneumoniae* AcrB transporter was generated by homology modelling using Swiss Model webserver, by applying Uniprot code Q93K40 as the amino acid sequence. The template was AcrB of *Escherichia coli*, with PDB ID code 4DX5 and resolution 1.9 Å. The sequence identity between the target and template was 91.6%. The accuracy and validity of the generated AcrB model was examined by Phyre2^60^ and the MPI Bioinformatics toolkit^61^. Phenylalanyl-arginine-β-naphthylamide (PAβN) and tetracycline (TET) PDB structures were generated by Chem3D 15.0. All the receptor and ligand structures were minimized by the SYBYL program.

### Molecular docking

The starting structures for running simulations were obtained by molecular docking of the ligands to the AcrB transporter. The orientation of the ligands within the multi-binding site of the binding protomer was taken from docking calculations performed with the AutoDock SMINA package^59^ through blind molecular docking, to find the cavity for ligands with the best affinity among all the probable ones. All the parameters were kept at their default values for running SMINA.

Then, an evaluated flexible molecular docking was performed using GOLD^56^ molecular docking into the SMINA-located binding site. The Genetic algorithm (GA) is used in GOLD ligand docking to examine the ligand conformational flexibility, along with the partial flexibility of the protein. The maximum number of runs was set to 20 for each compound, with the default parameters (100 population size, 5 for the number of islands, 100,000 number of operations and 2 for the niche size). Default cut off values of 2.5 Å (dH-X) for hydrogen bonds and 4.0 Å for van-der-Waals distance were used. When the solutions attained RMSD values within 1.5 Å, GA docking was terminated.

### Molecular dynamics (MD) simulations

In this stage, the method section includes (i) preparatory energy minimizations, heating the system, cMD equilibration, (ii) building a simulation environment suitable for aMD simulations.

cMD simulations were performed using the AMBER 12 package program^58^. A time step of 2 fs was used. Periodic boundary conditions were used, and electrostatic interactions were treated using the particle-mesh Ewald method, with a real-space cutoff of 12 Å and a grid spacing of 1 Å per grid point in each dimension. The van der Waals interactions were modeled with a Lennard–Jones potential, using a 12 Å cutoff. The simulations were performed in NPT ensemble, which means the temperature and pressure conditions were constant, and the temperature was kept at 300 K by applying the Langevin thermostat to all heavy atoms, with the Langevin damping constant set to 5 ps-1. The pressure was kept at 1 atm using the isotropic position scaling protocol used in AMBER.

The minimization was performed in two phases, and each phase was performed in two stages. In the first phase, ions and all water molecules were minimized for 2500 cycles of steepest descent followed by 2500 cycles of conjugate gradient minimization. Afterward, the entirety of the systems were minimized for a total of 10000 cycles without restraint, wherein 5500 cycles of steepest descent were followed by 4500 cycles of conjugate gradient minimization. After minimizations, the systems were heated for 500 ps while the temperature was raised from 0 to 300 K, and then equilibration was performed without a restraint for 1000 ps while the temperature was kept at 300 K. Sampling of reasonable configurations was conducted by running 100 ns simulations for each system.

Using the relaxed starting structure, obtained from cMD, the necessary information was calculated to set the necessary aMD parameters. Calculations of the added values for performing aMD simulations were shown at the end of the SI file as a note. From cMD run, we obtained the average total potential energy of −54,6820 and −59,5545 kcal/mol, and average dihedral energy of 32671 and 32605 kcal/mol for AcrB-PAβN and AcrB-TET systems, respectively. Using this information, and considering AcrB has 3093 residues and that the two mentioned complex systems have 196809 and 211425 atoms, respectively, the aMD parameters were calculated; Average total potential energy threshold (EthreshP) were obtained as −515,331 and −561,717 kcal/mol; Inverse strength boost factor for the total potential energy (alphaP) were obtained 31,489 and 33,828 kcal/mol; Average dihedral energy threshold (EthreshD) were obtained 45,047 and 44,961 kcal/mol; Inverse strength boost factor for the dihedral energy (alphaD) were obtained 2,475 and 2,471 kcal/mol, for AcrB-PAβN and AcrB-TET systems respectively. Then, the full 200 ns aMD simulations were run for each system.

### Postprocessing analyses: MM-PBSA/MM_GBSA calculation

The free energy of binding of the compounds to AcrB was evaluated by means of the molecular mechanic energies combined with the Poisson–Boltzmann and generalized Born and surface area continuum solvation (MM/PBSA/MM/GBSA) postprocessing methods^62,63^. In these method, the binding free energy of each compound is evaluated as

1 $${\Delta {\rm{G}}}_{{\rm{bind}}}={{\rm{G}}}_{{\rm{com}}}-{{\rm{G}}}_{{\rm{rec}}}+{{\rm{G}}}_{{\rm{lig}}}$$

with G_com_, G_rec_, and G_lig_ being the absolute free energies of complex, receptor, and ligand, respectively, averaged over the equilibrium trajectory of the complex (single-trajectory approach). According to these schemes, the free-energy difference can be decomposed as

2 $${\Delta {\rm{G}}}_{{\rm{bind}}}={\Delta {\rm{E}}}_{{\rm{MM}}}+{\Delta {\rm{G}}}_{{\rm{solv}}}-{{\rm{T}}\Delta {\rm{S}}}_{{\rm{conf}}}$$

where ΔE_MM_ is the difference in the molecular mechanics energy, ΔG_solv_ is the solvation-free energy, and ΔS_conf_ is the solute conformational entropy. The first two terms were calculated with the following equations:

3 $${\Delta {\rm{E}}}_{{\rm{MM}}}={\Delta {\rm{E}}}_{{\rm{bond}}}+{\Delta {\rm{E}}}_{{\rm{angle}}}+{\Delta {\rm{E}}}_{{\rm{torsion}}}+{\Delta {\rm{E}}}_{{\rm{vdw}}}+{\Delta {\rm{E}}}_{{\rm{elec}}}$$

4 $${\Delta {\rm{G}}}_{{\rm{solv}}}={\Delta {\rm{G}}}_{\mathrm{solv},{\rm{p}}}+{\Delta {\rm{G}}}_{\mathrm{solv},{\rm{np}}}$$

E_MM_ includes the molecular mechanics energy contributed by the bonded (Ebond, Eangle, and E_torsion_) and nonbonded (E_vdw_ and E_ele_, calculated with no cutoff) terms of the force field. ΔG_solv_ is the solvation-free energy, which can be modeled as the sum of an electrostatic contribution (ΔG_solv,p_, evaluated using the MMGBSA or MM-PBSA approach) and a nonpolar one (ΔG_solv,np_ = γΔSA + b, proportional to the difference in solvent-exposed surface area, ΔS_A_).

In this approach for the fully equilibrated structures (Fig. S6), 20 snapshots were collected from the last 200 ps of cMD simulations of complex systems for post processing analysis of free energy calculation. The RMSD of the ligands (Fig S7) with respect to the last conformation of the corresponding ligands sampled during the MD were calculated which showed the ligands in complex with the systems reached steady state at the end of the simulation The ΔG_PB_ term was calculated by solving the finite-difference Poisson-Boltzmann equation using the internal PBSA program. The SCALE value was set to 2. The Parse radii were employed for all atoms^64^. The solvent probe radius was set at 1.4 Å (with the radii in the prmtop files). MM-PBSA running was performed with the pbsa module (PROC = 2). The value of the exterior dielectric constant was set at 80, and the solute dielectric constant was set at 1^65^. The nonpolar contribution was determined on the basis of the solvent accessible surface area (SASA) using the LCPO method^66^ and CAVITY-OFFSET set at 0.00.

Finally, the conformational entropy contribution, to estimate the absolute binding free energy, was calculated through normal-mode analysis using the ‘nmode’ module of AMBER.

### Postprocessing analyses: Analysis of protein conformational change using principal component analysis (PCA)

The original trajectory files produced by aMD were significantly large, and we can’t include them to Bio3d package installed in R program due to space limitations, so the trajectories in each aMD simulations were down sampled with an interval of 100. The points represented in the conformer plots were computationally clustered and colored by the cluster. This was performed by creating a distance matrix of the principal components of interest.

PCA reduces the dimensionality of large data sets by calculating a covariance matrix and its eigenvectors. Vectors with the highest eigenvalues become the most significant principal components. When principal components are plotted against each other, similar structures cluster. Each cluster then theoretically shows a different protein conformational state. To avoid sample noise from random fluctuations^48,67^, following aMD simulations of the AcrB transporter, the PCA was calculated only for Cα atoms. Then, each protomer was selected in a separate PCA analysis, which was a good discriminator of conformations.

With the Bio3D package installed in R, the plot command has been overloaded to create a default PCA plot with four graphs. Three are the z-scores of the first three principal components plotted against each other in two dimensions. The last is a scree plot showing how much of the variance of the data set is captured by each principal component.

### Data availability

All data generated or analysed during this study are included in this published article (and its Supplementary Information files).

## Electronic supplementary material

Supporting Information

Supplementary Video 1

Supplementary Video 2

Supplementary Video 3

Supplementary Video 4
