# Author Correction: Mapping the Dynamic Functions and Structural Features of AcrB Efflux Pump Transporter Using Accelerated Molecular Dynamics Simulations

**DOI:** 10.1038/s41598-019-54251-6

**Published:** 2019-11-28

**Authors:** Shirin Jamshidi, J. Mark Sutton, Khondaker Miraz Rahman

**Affiliations:** 1 School of Cancer and Pharmaceutical Science, King’s College London, London, SE1 9NH UK; 2 Public Health England, National Infection Service, Porton Down, Salisbury, Wiltshire SP4 0JG UK

Correction to: *Scientific Reports* 10.1038/s41598-018-28531-6, published online 11 July 2018

Supplementary files containing Movies S1 – S4 were omitted from the original version of this Article. This has been corrected in the HTML version of the Article; the PDF was correct at the time of publication.

