# Supplementary material for: Mapping the Dynamic Functions and Structural Features of AcrB Efflux Pump Transporter Using Accelerated Molecular Dynamics Simulations
Source: Sci Rep. 2018 Jul 11;8:10470. doi: 10.1038/s41598-018-28531-6 (PMC6041327; doi:10.1038/s41598-018-28531-6)
Supplement: Supplementary file 1 — Supporting Information [file 41598_2018_28531_MOESM1_ESM.docx]

**Supporting Information**

**Mapping the Dynamic Functions and Structural Features of AcrB Efflux Pump Transporter Using Accelerated Molecular Dynamics Simulations**

Shirin Jamshidi^a^, J. Mark Sutton^b^, Khondaker Miraz Rahman^a*^

^a^ School of Cancer and Pharmaceutical Science, King’s College London, London, SE1 9NH, UK

^b^ Public Health England, National Infection Service, Porton Down, Salisbury, Wiltshire, SP4 0JG, UK.

^*^To whom correspondence should be addressed. [k.miraz.rahman@kcl.ac.uk](mailto:k.miraz.rahman@kcl.ac.uk)

**Supplementary Figures and Tables**

^
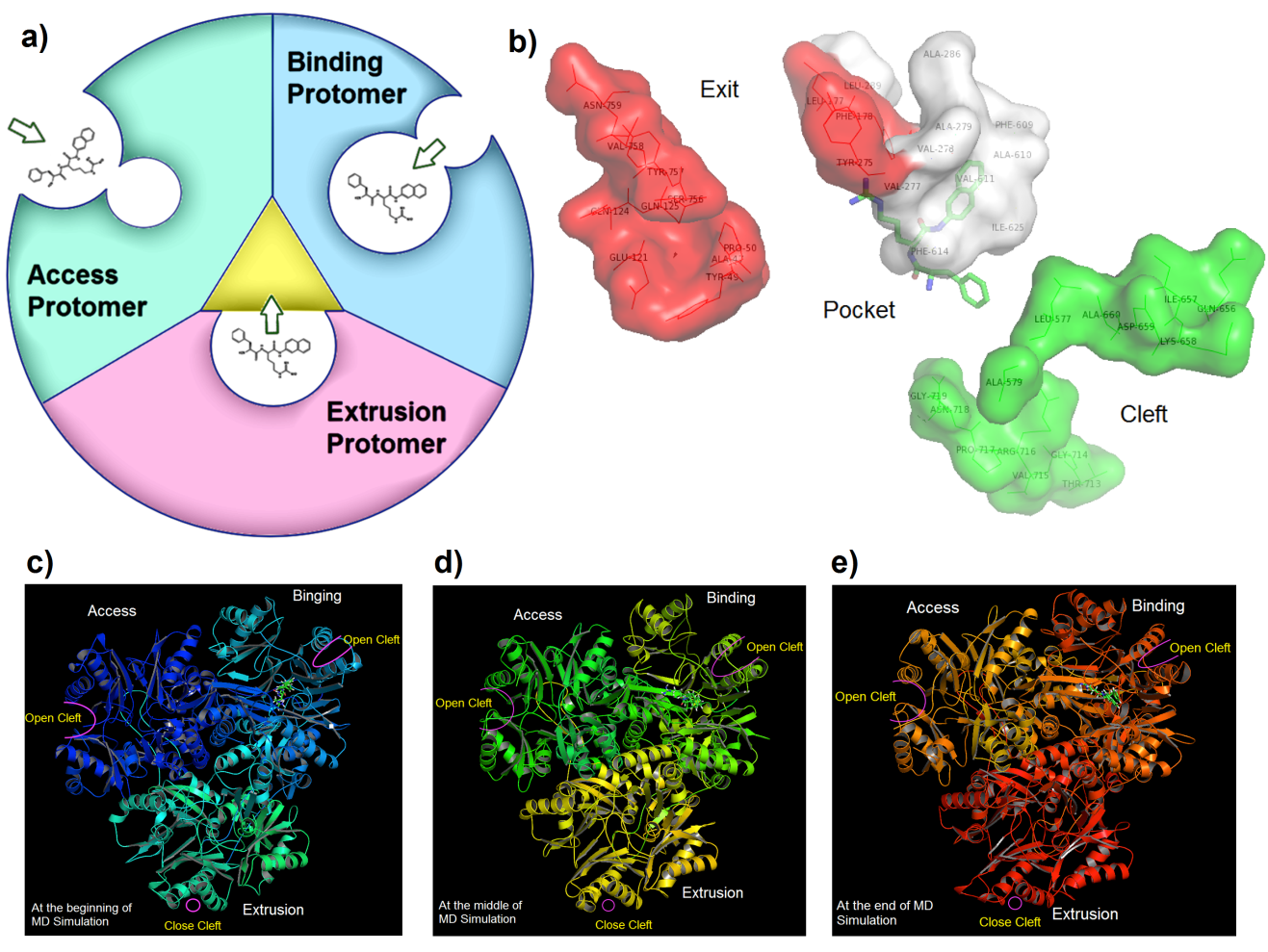
^

Fig S1. a) Schematic top view of AcrB in interaction with PAβN. The displacement process of the ligand from the multi-binding site of the transporter confers different conformational changes in the functionally diverse monomers of the trimer structure of the efflux pump. Each colour represents a different stage in the protomer of the transporter, and the central cavity channel is yellow. b) Key residues in exit port (red), binding pocket (gray) and cleft (green) in side view of the transporter.

**a)**

**
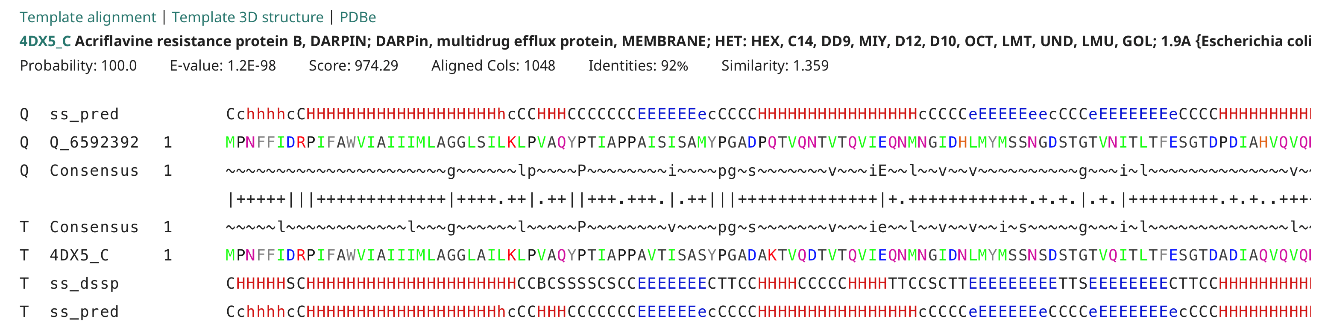
**

**b)**

**
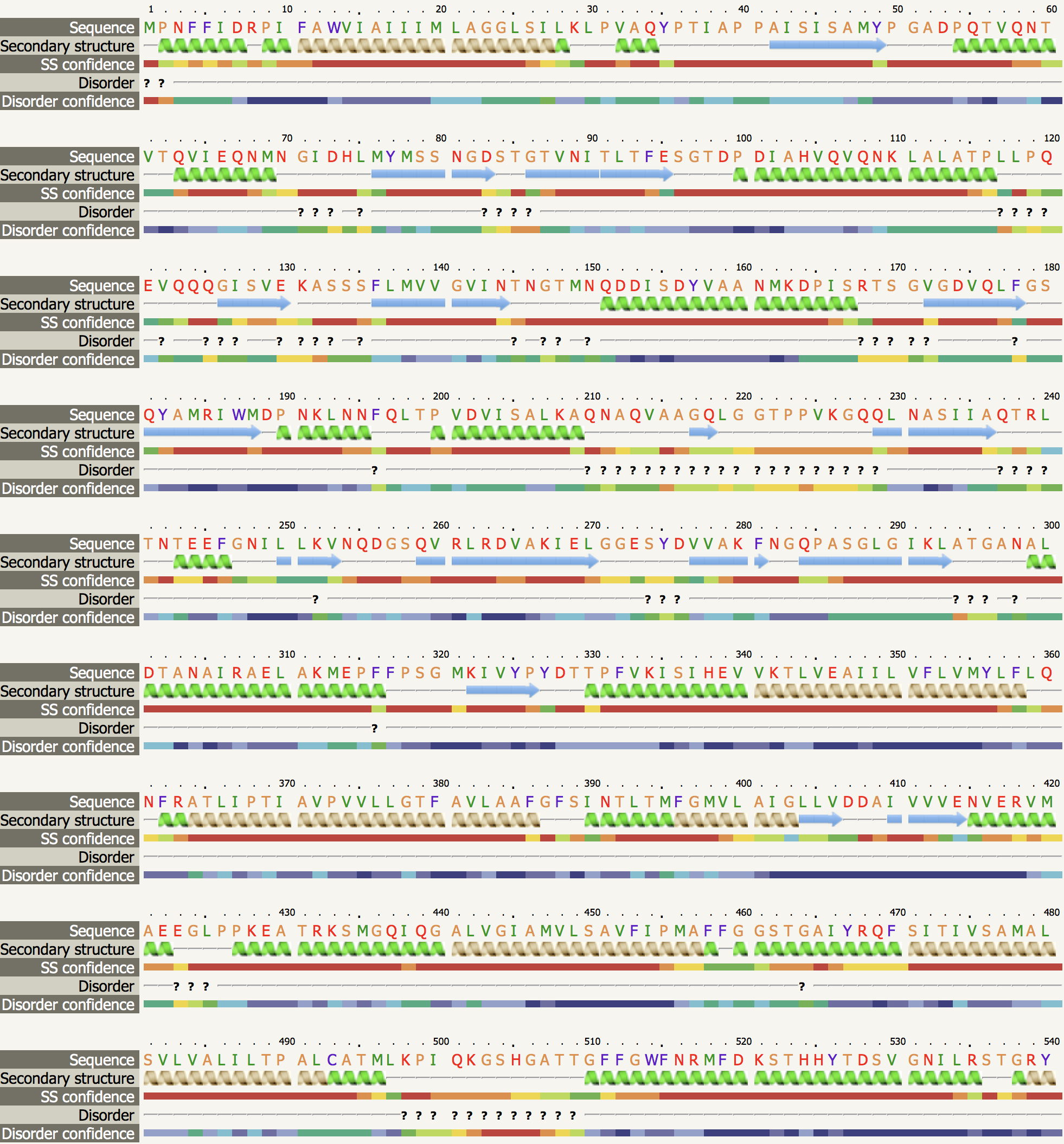
**

**
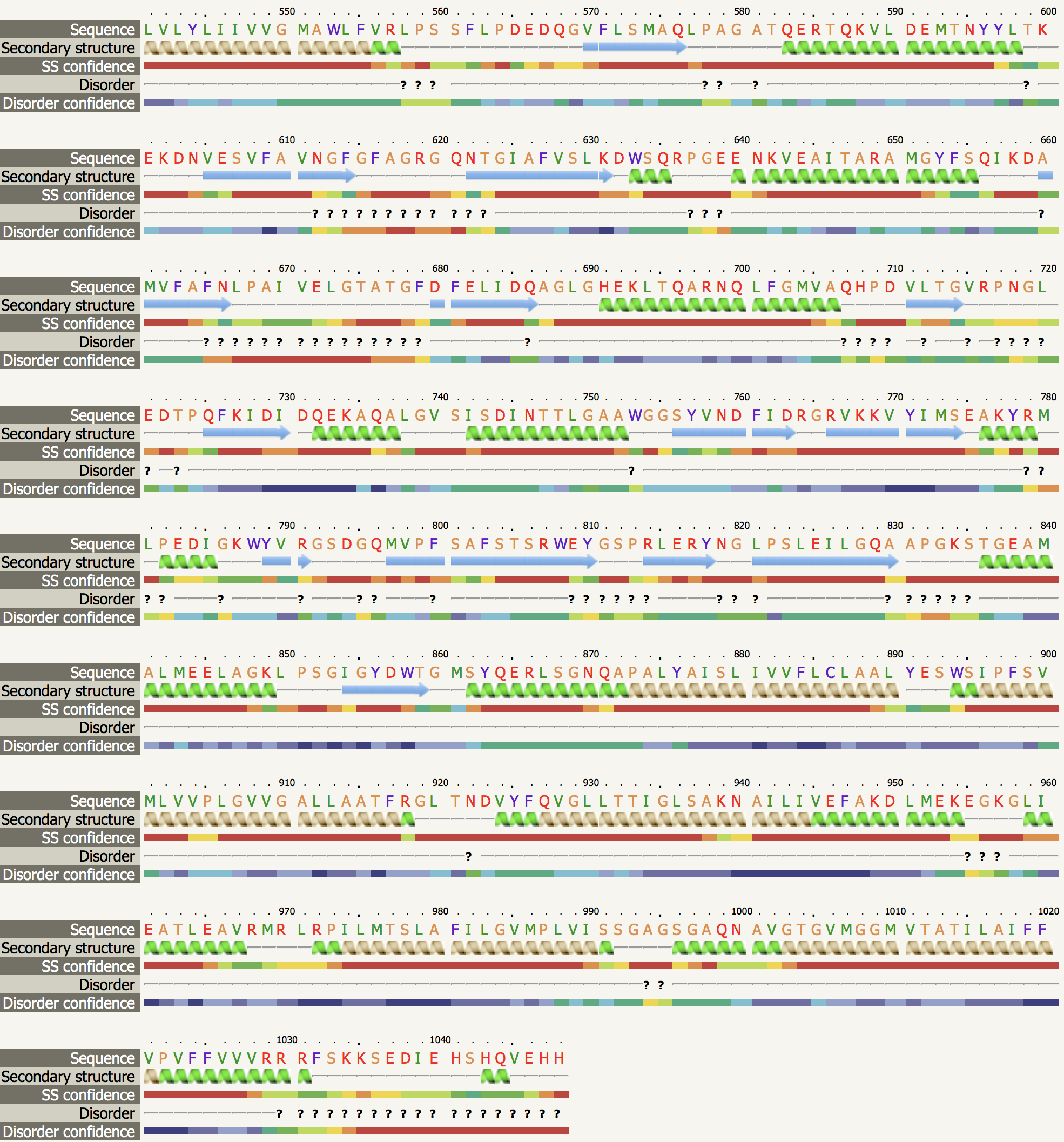

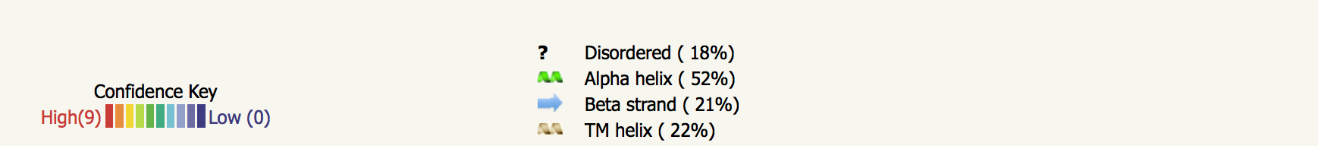
**

**c)**

**
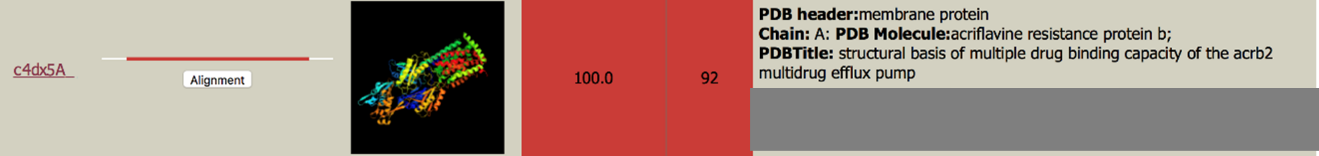
**

**Fig S2.** The validation of the structural model for AcrB model generated with template AcrB from *Escherichia Coli* (PDB ID code 4DX5); a) HHpred A) HHpred b) Secondary structure and disorder prediction c) Fold recognition. Phyre2 webserver and Max-planck Bioinformatic Toolkit were applied to prepare this data.

http://www.sbg.bio.ic.ac.uk/phyre2/html/page.cgi?id=index

<https://toolkit.tuebingen.mpg.de>


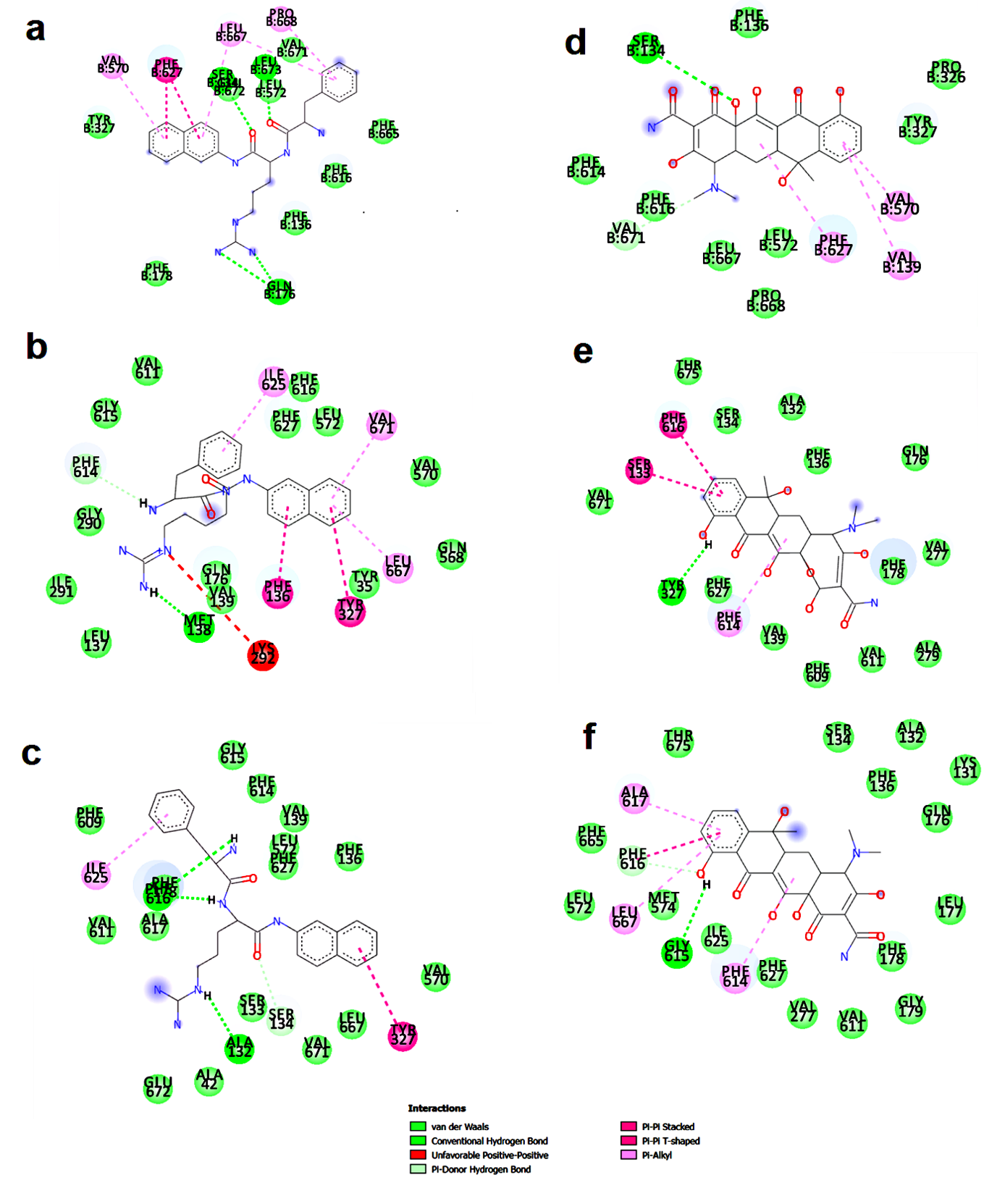


**Fig S3.** 2D structures of PAβN (left column) and tetracycline (right column) in the multi binding site of AcrB; a) after GOLD molecular docking, b) average structure after 100 ns cMD and c) average structure after 200 ns aMD.


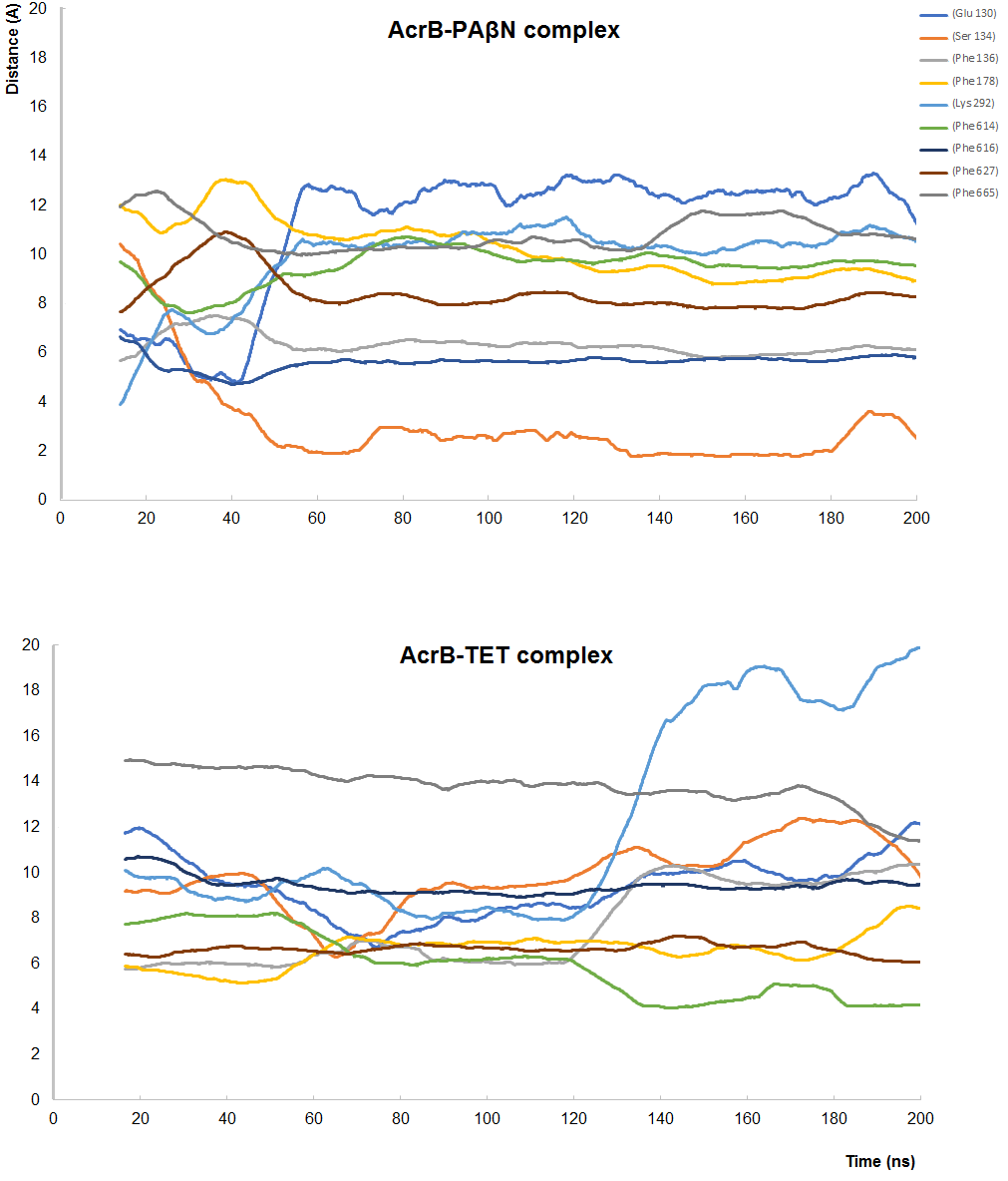


**Fig S4.** Trendlines of the distances between the ligands and some key residues of the multi-binding site in the course of 200 ns aMD simulations.


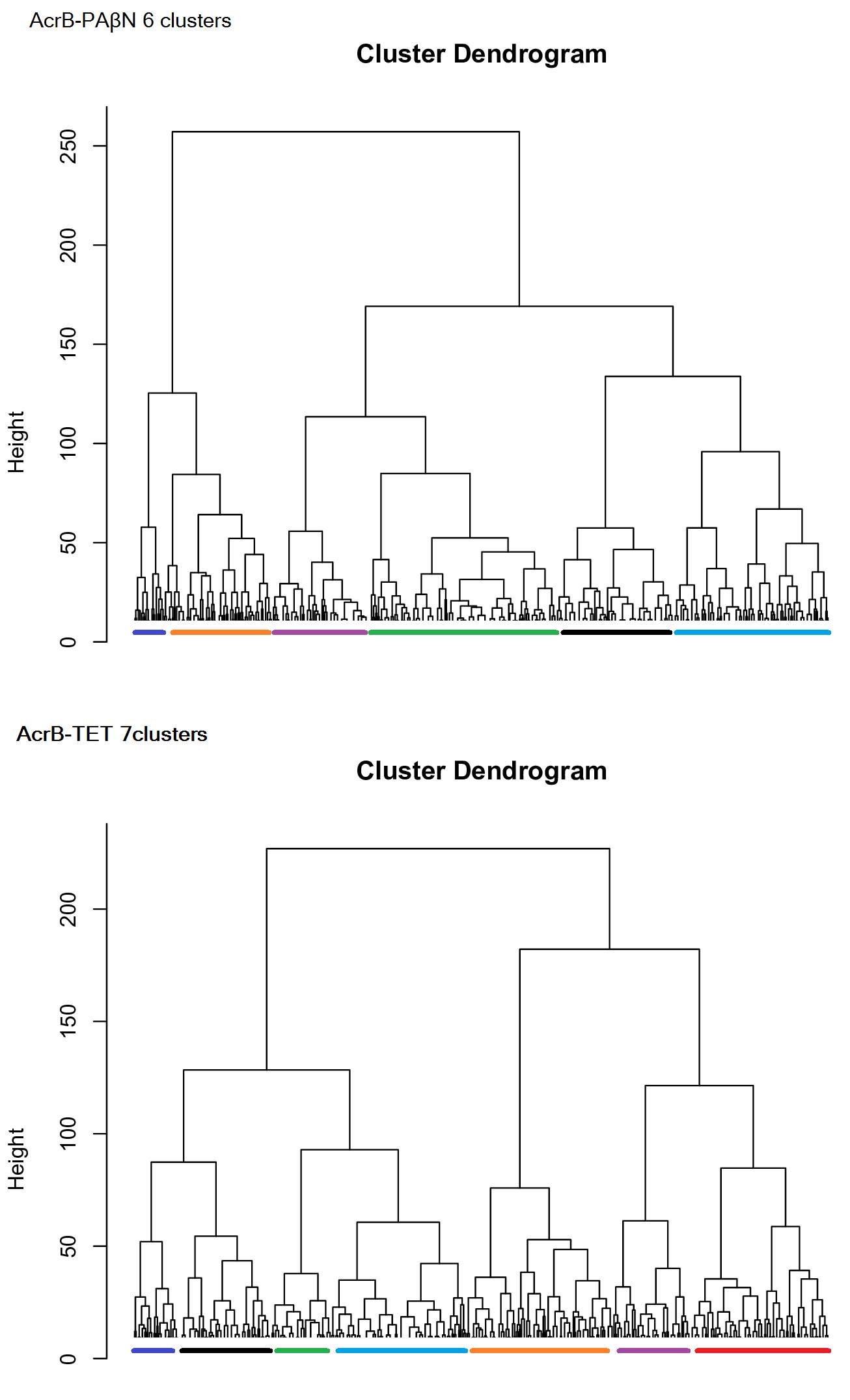


**Fig S5.** RMSD clustering of AcrB structures; The dendrogram data appeared to fall into six clusters for AcrB-PAβN complex, and seven clusters for AcrB-TET complex. Each cluster has been colored to demonstrate that they belong to a separate group.


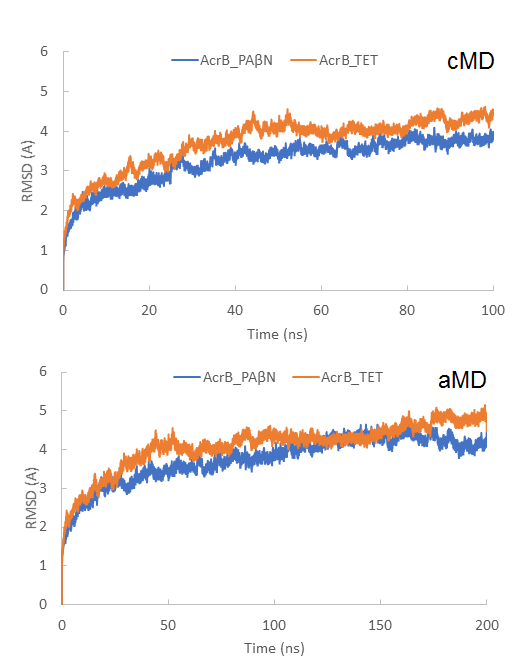


**Fig S6.** Time dependence of root-mean-square deviation (RMSD) (Å) of AcrB for the backbone atoms in the MD simulations of both complexes.


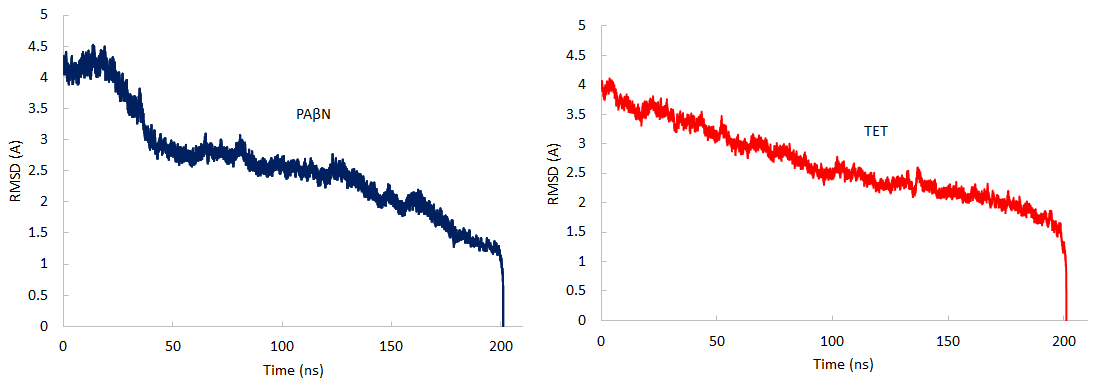


**Fig S7.** Ligands’ RMSD with respect to the last conformation of the corresponding ligands sampled during the MD

**Table S1.** Interaction energies between ligands and responsive amino acid residues with favorable interaction energy in the extracted structures from cMD simulation trajectories in each 10 ns. The common amino acids are in different panels, which show the different times in MD simulations (bolded).

|  | **AcrB-PAβN** | **AcrB-TET** |
| --- | --- | --- |
|  | **Residue / No. / Interaction**  **energy (kj/mol)** | **Residue / No. / Interaction**  **energy (kj/mol)** |
| 10 ns | Arg 619 -7.48  Ile 625 -13.4  **Phe 136 -12.7**  Phe 614 -10.9  **Phe 616 -17.0**  **Phe 627 -21.5**  Tyr 327 -8.2 | Phe 178 -22.0  **Phe 614 -16.0**  Phe 616 -10.0  Phe 627 -8.4  Ser 134 -10.0  Tyr 327 -9.8 |
| 20 ns | Ala 132 -13.5  Gln 176 -9.9  **Phe 136 -29.6**  Phe 614 -9.7  **Phe 616 -40.6**  **Phe 627 -8.4**  Ser 134 -10.1 | Gln 176 -11.0  Phe 178 -8.3  **Phe 614 -26.0**  Phe 627 -9.2  Ser 133 -11.0  Ser 134 -10.0  Val 611 -8.3 |
| 30 ns | Leu 667 -8.1  **Phe 136 -19.3**  Phe 178 -8.5  **Phe 616 -34.9**  **Phe 627 -8.2**  Ser 133 -10.0  Ser 134 -18.9 | Phe 614 -13.0  Phe 616 -14.0  Phe 627 -12.0  Ser 133 -11.0 |
| 40 ns | Leu 667 -8.1  **Phe 136 -19.3**  Phe 178 -8.5  Phe 614 -6.7  **Phe 616 -34.9**  **Phe 627 -8.2**  Ser 133 -10.0  Ser 134 -18.9 | **Phe 614 -35**.0  Phe 627 -9.7  Val 277 -9.1  Val 611 -7.1 |
| 50 ns | Ala 132 -11.5  **Phe 136 -16.9**  Phe 614 -7.2  **Phe 616 -22.3**  **Phe 627 -7.3**  Ser 134 -19.9 | Ala 132 -8.2  Phe 178 -14.0  Phe 614 -34.0  Phe 616 -11.0  Phe 627 -7.1 |
| 60 ns | Ala 132 -11.7  **Phe 136 -19.5**  Phe 178 -8.1  **Phe 616 -35.8**  **Phe 627 -9.4**  Ser 133 -8.7  Ser 134 -18.5 | Ala 132 -8.2  **Phe 614 -34**.0  Phe 616 -11.0  Phe 627 -7.1 |
| 70 ns | Ala 132 -7.7  **Phe 136 -21.8**  Phe 178 -12.5  **Phe 616 -39**.0  **Phe 627 -12.4**  Ser 134 -19.9  Tyr 327 -7.9 | Ala 132 -8.2  Phe 178 -14.0  **Phe 614 -34**.0  Phe 616 -11.0  Phe 627 -7.1 |
| 80 ns | Ala 132 -8.1  Ala 617 -8.4  **Phe 136 -14.1**  Phe 178 -8.9  **Phe 616 -35.3**  **Phe 627 -11.8**  Ser 133 -9.6  Ser 134 -20 | Ala 132 -8.2  Phe 178 -14.0  Phe 614 -34.0  Phe 616 -11.0  Phe 627 -7.1 |
| 90 ns | Ala 132 -8.9  Ala 617 -14.6  Leu 667 -7.8  **Phe 136 -22.2**  Phe 178 -8.7  **Phe 616 -31.8**  **Phe 627 -10.1**  Ser 134 -16.7  Tyr 327 -7.2 | Ala 132 -8.2  Phe 178 -14.0  **Phe 614 -34**.0  Phe 616 -11.0  Phe 627 -7.1 |
| 100 ns | Ala 132 -9.8  Ile 625 -7.6  **Phe 136 -9.6**  Phe 178 -12.4  **Phe 616 -26.2**  **Phe 627 -18.6**  Ser 133 -8.5  Ser 134 -18.3  Tyr 327 -8.2 | Ala 617 -13.0  Ile 625 -7.0  Phe 178 -20.0  **Phe 614 -33**.0 Phe 616 -13.0 |

**Table S2.** Interaction energies between ligands and responsive amino acid residues in the average extracted structures from aMD simulation trajectories. The data indicates that Ala132, Ala617, Ser134, Phe627, Phe136 and Phe616 in AcrB-PAβN complex, and Phe614, Phe178 and Phe616 in AcrB-TET complex interact effectively with each ligand during the time of aMD. Since these amino acid residues are implicated in hydrogen bond and/or Pi-cation interactions, the data consequently show that they are key residues involved in association and effective interaction between the ligands and porter.

| **AcrB-PAβN** | | | **AcrB-TET** | | |
| --- | --- | --- | --- | --- | --- |
| **Residue** | **No.** | **Interaction**  **energy (kj/mol)** | **Residue** | **No.** | **Interaction**  **energy (kj/mol)** |
| Ala | 42 | -0.5 | Ala | 132 | -2.3 |
| Ala | 132 | -9.9 | Ala | 617 | -1.7 |
| Ala | 617 | -8.5 | Gln | 176 | -2.1 |
| Arg | 619 | 1.0 | Gly | 179 | -0.5 |
| Arg | 716 | 1.0 | Gly | 615 | -1.1 |
| Arg | 814 | 0.8 | Ile | 625 | -4.0 |
| Asp | 101  174 | -0.3 | Leu | 177 | -3.1 |
| Asp |  | -1.2 | Leu | 572 | -0.4 |
| Asp | 680 | -1.6 | Phe | 136 | -1.8 |
| Gln | 176 | -0.3 | Phe | 178 | -25.6 |
| Glu | 130 | -1.6 | Phe | 614 | -39.2 |
| Glu | 672 | -1.7 | Phe | 616 | -9.8 |
| Glu | 682 | -1.0 | Phe | 627 | -6.8 |
| Glu | 816 | -0.7 | Ser | 133 | -0.3 |
| Glu | 825 | -0.8 | Ser | 134 | -3.2 |
| Gly | 615 | -4.5 | Thr | 675 | -1.5 |
| Gly | 618 | -0.3 | Val | 277 | -3.4 |
| Ile | 625 | -6.2 | Val | 611 | -4.4 |
| Leu | 572 | -0.5 | Val | 671 | -3.2 |
| Leu | 667 | -4.4 |  |  |  |
| Lys | 110 | 0.3 |  |  |  |
| Lys | 131 | 0.9 |  |  |  |
| Lys | 163 | 0.4 |  |  |  |
| Lys | 292 | 2.2 |  |  |  |
| Lys | 642 | 0.3 |  |  |  |
| Lys | 769 | 0.3 |  |  |  |
| Phe | 136 | -20.6 |  |  |  |
| Phe | 178 | -7.2 |  |  |  |
| Phe | 614 | -6.5 |  |  |  |
| Phe | 616 | -29.9 |  |  |  |
| Phe | 627 | -13.7 |  |  |  |
| Ser | 133 | -6.6 |  |  |  |
| Ser | 134 | -13.8 |  |  |  |
| Tyr | 327 | -7.9 |  |  |  |
| Val | 139 | -1.5 |  |  |  |
| Val | 570 | -0.7 |  |  |  |
| Val | 671 | -5.0 |  |  |  |
| Lys | 110 | 0.4 |  |  |  |

**Table S3.** The most favorable interaction energies between ligands and responsive amino acid residues in the average extracted structures from aMD simulations trajectories.

| **AcrB-PaβN** | | | **AcrB-TET** | | |
| --- | --- | --- | --- | --- | --- |
| **Residue** | **No.** | **Interaction**  **energy (kj/mol)** | **Residue** | **No.** | **Interaction**  **energy (kj/mol)** |
| Ala | 132 | -9.9 | Phe | 178 | -25.6 |
| Ala | 617 | -8.5 | Phe | 614 | -39.2 |
| Phe | 136 | -20.6 | Phe | 616 | -9.8 |
| Phe | 178 | -7.2 |  |  |  |
| Phe | 616 | -29.9 |  |  |  |
| Phe | 627 | -13.7 |  |  |  |
| Ser | 134  327 | -13.8 |  |  |  |
| Tyr |  | -7.9 |  |  |  |

**Table S4.** Calculated energy contributions to form the AcrB−PAβN and AcrB−TET complexes (kcal/mol) and inhibition constants (K_d_ in Molar) with standard errors of the mean (in parentheses) after a) cMD and b) aMD.

1. cMD

| Energy distributions | AcrB-PAβN | AcrB-TET |
| --- | --- | --- |
| ΔE_ele_ | -16.7 (2.1) | -20.1 (2.2) |
| ΔE_vdw_ | -49.2 (2.0) | -41.6 (1.1) |
| ΔE_sol_ | 37.8 (3.3) | 49.7 (3.5) |
| ΔG_PB_ | -28.1 (2.9) | -11.9 (1.9) |
| ΔG_GB_ | -35.9 (2.3) | -16.3 (2.3) |
| -TΔS | 18.7 | 18.9 |
| ΔG_bind_ | -9.4 (0.7) | 6.9 (0.5) |
| K_d_ ^*^ | 1.4×10^-7^ | 1.2×10^5^ |
| K_d_ (Bulk)^**^ | 8.4×10^17^ | 7.3×10^29^ |

1. aMD

| Energy distributions | AcrB-PAβN | AcrB-TET |
| --- | --- | --- |
| ΔE_ele_ | -22.1 (4.4) | -27.1 (3.7) |
| ΔE_vdw_ | -54.6 (2.9) | -46.3 (1.6) |
| ΔE_sol_ | 36.1 (4.5) | 49.1 (4.6) |
| ΔG_PB_ | -40.6 (3.5) | -24.4 (2.7) |
| ΔG_GB_ | -41.5 (2.6) | -23.6 (2.7) |
| -TΔS | 17.6 | 19.5 |
| ΔG_bind_ | -23.1 (2.3) | -4.9 (0.4) |
| K_d_ ^*^ | 1.5×10^-17^ | 2.6×10^-6^ |
| K_d_ (Bulk)^**^ | 0.9×10^-8^ | 1.6×10^21^ |

*Kd obtained by using ΔG = RT ln K_d_ formula

**Calculated by considering Avogadro’s number

**Movie S1.** Visualization of the fluctuations captured by the first principal component of the PAβN-bound AcrB transporter as a repeated trajectory movie, generated by PyMOL.

**Movie S2.** Visualization of the fluctuations captured by the first principal component of the TET-bound AcrB transporter as a repeated trajectory movie, generated by PyMOL.

**Movie S3.** Visualization of the aMD trajectories of the PAβN-bound AcrB transporter by VMD and VideoMach.

**Movie S4.** Visualization of the aMD trajectories of the TET-bound AcrB transporter by VMD and VideoMach.
